# Altered Pain in the Brainstem and Spinal Cord of Fibromyalgia Patients During the Anticipation and Experience of Experimental Pain

**DOI:** 10.3389/fneur.2022.862976

**Published:** 2022-05-06

**Authors:** Gabriela Ioachim, Howard J. M. Warren, Jocelyn M. Powers, Roland Staud, Caroline F. Pukall, Patrick W. Stroman

**Affiliations:** ^1^Center for Neuroscience Studies, Queen's University, Kingston, ON, Canada; ^2^Department of Medicine, University of Florida, Seffner, FL, United States; ^3^Department of Psychology, Queen's University, Kingston, ON, Canada; ^4^Department of Biomedical and Molecular Sciences, Queen's University, Kingston, ON, Canada; ^5^Department of Physics, Queen's University, Kingston, ON, Canada

**Keywords:** fMRI, brainstem, spinal cord, pain, human, chronic, fibromyalgia

## Abstract

Chronic pain associated with fibromyalgia (FM) affects a large portion of the population but the underlying mechanisms leading to this altered pain are still poorly understood. Evidence suggests that FM involves altered neural processes in the central nervous system and neuroimaging methods such as functional magnetic resonance imaging (fMRI) are used to reveal these underlying alterations. While many fMRI studies of FM have been conducted in the brain, recent evidence shows that the changes in pain processing in FM may be linked to autonomic and homeostatic dysregulation, thus requiring further investigation in the brainstem and spinal cord. Functional magnetic resonance imaging data from 15 women with FM and 15 healthy controls were obtained in the cervical spinal cord and brainstem at 3 tesla using previously established methods. In order to investigate differences in pain processing in these groups, participants underwent trials in which they anticipated and received a predictable painful stimulus, randomly interleaved with trials with no stimulus. Differences in functional connectivity between the groups were investigated by means of structural equation modeling. The results demonstrate significant differences in brainstem/spinal cord network connectivity between the FM and control groups which also correlated with individual differences in pain responses. The regions involved in these differences in connectivity included the LC, hypothalamus, PAG, and PBN, which are known to be associated with autonomic homeostatic regulation, including fight or flight responses. This study extends our understanding of altered neural processes associated with FM and the important link between sensory and autonomic regulation systems in this disorder.

## Introduction

Fibromyalgia (FM) is a chronic pain condition that is characterized by both hyperalgesia (heightened pain sensitivity) and allodynia (disproportionate pain or sensitivity from sensory stimuli that would not normally be painful) (1–3). Most evidence to date suggests that the abnormal pain responses in FM may be the result of central sensitization (4–11), which has prompted functional magnetic resonance imaging (fMRI) studies of the central nervous system. However, the majority of these studies focus on the brain (8, 12–20) and a large proportion used model-driven analyses, which we have recently shown may provide an incomplete picture when investigating pain processing with fMRI (21, 22). Importantly, additional studies examining the brainstem and spinal cord, which include regions that are known to play key roles in descending pain modulation (23), can advance knowledge of pain processing in FM.

Most prior MRI studies of FM in the brainstem and spinal cord included structural studies (24, 25) and studies of resting-state function which did not involve a painful stimulus (26). However, functional studies that involved noxious stimuli have also been carried out, and have provided crucial evidence of altered pain processing. The results have demonstrated differences in BOLD signal changes associated with temporal summation of pain and descending modulation in women with fibromyalgia compared to healthy controls (7, 11). These studies, however, modeled the time course of neuronal activation only during and after noxious stimulation.

Our recent study has demonstrated that pain modulation in the brainstem and spinal cord includes both a reactive component and a continuous component of pain modulation related to cognitive and emotional influences on pain, which are present before, during, and after a painful stimulus (27). Brainstem and spinal cord network connectivity variations have been described both before and during stimulation in healthy volunteers, and they appear to be related to pain expectation or pain relief in (28, 29). Some of these effects related to pain expectations may also be linked in part to autonomic homeostatic regulation in a subset of brainstem regions (28, 30). Previous evidence shows that functional differences in FM exist in brain regions linked to motivational-affective components of pain processing (12–15). Therefore it is possible that similar important differences in pain modulation may also exist in brainstem and spinal cord regions. Some behavioral studies have linked changes in autonomic regulation to changes in pain sensitivity in FM (31–33), but this has not been investigated with functional neuroimaging studies.

The objective of the present study was to advance our understanding of the neural processes underlying heighted pain sensitivity in FM, by means of fMRI in the brainstem and spinal cord, to investigate function during both the anticipation and experience of pain. We used structural equation modeling to investigate a network of brainstem and spinal cord regions associated with descending pain modulation (23), motivational-affective components of pain (28, 29) and autonomic homeostatic regulation (34). We hypothesized that during the anticipation and experience of pain, the connectivity in spinal cord and brainstem networks was altered in FM compared to healthy controls.

## Methods

All study methods were reviewed and approved by our institutional research ethics board, and participants provided fully informed written consent before participating. The study protocol conformed to the ethical guidelines of the 2013 Declaration of Helsinki.

### Participant Recruitment

Participants with and without FM were recruited through online advertisements as well as physical flyers posted in the general community and in chronic pain support groups. Participation involved two fMRI sessions as part of a larger study, one imaging the brain and the other imaging the brainstem and spinal cord, although not all participants completed both sessions. The current study of the brainstem and spinal cord involved 15 women with FM (mean age 46 ±13 years) who fulfilled the 1990 and 2016 FM criteria, and 15 healthy women (mean age 39 ±10 years). All participants were free of any contraindications for MR imaging (e.g., metallic implants, claustrophobia, pregnancy, etc.), were not taking any centrally-acting medications. They were allowed to continue on other medications if they were taking them for at least 3 months prior to the study. The participants were not asked to stop medication they were already taking, as reports suggest that conventional treatments do not alleviate fibromyalgia pain, and stopping medications may pose a risk to a participant's health. Participants taking centrally-acting pain medication, however, were still excluded from the study.

### Questionnaires

In addition to the imaging data, participants completed a series of questionnaires related to demographic information, mental health, pain symptoms, and autonomic functioning. All participants completed the 2016 Fibromyalgia Survey Questionnaire (FSQ) (35) to assess whether they met the most recent classification criteria for FM, as some participants had been diagnosed over a decade previously by their physicians. Some studies have also found discrepancies between physician diagnoses of FM and classification based on the most recent diagnostic criteria (36). Participants also completed the State-Trait Anxiety Inventory (STAI) (37), and Beck Depression Inventory (BDI) (38), because FM has been associated with high anxiety and depression (1). The Social Desirability Scale (SDS) (39) and the Pain Catastrophizing Scale (PCS) (40) were also included to assess whether individual reports of pain ratings were associated with the desire to perform well for the study or the tendency to catastrophize painful sensations. The Composite Autonomic Symptom Score 31 (COMPASS-31) (41) was used to assess autonomic health. This questionnaire includes subscales for 6 domains of autonomic symptom severity, namely orthostatic intolerance, vasomotor, secretomotor, gastrointestinal, bladder, and pupillomotor symptoms. To assess pain and pain symptoms, we also included the Revised Fibromyalgia Impact Questionnaire (FIQR) (42) and the Short-Form McGill Pain Questionnaire-2 (SF-MPQ-2) (43). Participants in the HC group were still given the FIQR but the word “fibromyalgia” was omitted (questions referred to how pain impacted their lives) as these participants did not have any experience of fibromyalgia. The SF-MPQ-2 included four subscales of pain quality, namely affective descriptors, continuous, intermittent, and predominantly neuropathic pain. These questionnaire scores were used to compute group means for the FM and HC participants, which were then compared using *t*-tests. Group means for each subscale (in inventories that included subscales) were also computed and tested. Significant differences in average scores were inferred at a threshold of *p* < 0.05.

### Participant Training

All participants completed a 1-h sham training session before their imaging session. This time was used to familiarize the participant with the study paradigm, complete the algometry testing portion of the study, ease any anxiety about the imaging session by practicing in a sham MRI, and introduce them to the pain stimulus used in the study. This training session was the first time the participants were exposed to the numerical pain rating scale used in the study and the heat stimulus used. All sessions were carried out by two examiners to facilitate the training, one male and one female researcher. First, participants underwent tender point test according to the 1,190 ACR FM criteria (35, 44, 45). For simplicity and participant comfort, only 12 points above the waist were examined for pain (bilateral occiput, bilateral epicondyle, bilateral low cervical, bilateral supraspinatus, bilateral trapezius, and bilateral second rib) alongside a control point on the forehead (44, 45). For each point, a researcher applied pressure in even increments of 1 kg/s (to a maximum of 4 kg) with an algometer (FPK 10 pain test algometer, Wagner Instruments, Greenwich, Connecticut). Participants were instructed to say “stop” as soon as the sensation became painful, and the pressure needed to reach this point was recorded. If a participant did not report pain for a point even after the maximum pressure was reached, “no pain” was recorded. Each point was probed only once, and all pressure-point exams were conducted by the same researcher (male) for consistency in application.

Next, participants were introduced to the numerical pain intensity scale (NPS) they would use to rate their pain (46), as well as the stimulus used during the study. This scale ranges from 0 to 100 in increments of 10, with descriptors at each increment (0 = no sensation, 10 = warm, 20 = a barely painful sensation, 30 = very weak pain, 40 = weak pain, 50 = moderate pain, 60 = slightly strong pain, 70 = strong pain, 80 = very strong pain, 90 = nearly intolerable pain, 100 = intolerable pain). Participants were told they would not be experiencing temperatures that could cause harm to their skin, and that the study did not aim to induce pain above a rating of moderately severe (70) on the NPS.

This study used a MRI-compatible Robotic Contact-Heat Thermal Heat Stimulator (RTS-2) to deliver the noxious stimulus. A heat stimulus was chosen in order to compare these results to recent pain research in the spinal cord (7, 28, 47–49), as well as the fact that fibromyalgia has been associated with higher heat pain sensitivity (1). The RTS-2's plexiglass casing houses a heated aluminum thermode which can be advanced to exit the casing and touch the skin of a participant or retracted into the casing. The movement and temperature of the thermode are precisely controlled by custom written software in MATLAB (Mathworks Inc., Natick, MA). Participants were instructed to place their right hand on the casing so the thermode would make contact with the thenar eminence of their right hand. This placement was chosen because that area of the skin corresponds to the C6 dermatome and would allow these results to be compared to previous spinal cord studies of pain (27, 28, 48, 49). A number of calibration tests were performed to allow the participant to become familiar with the stimulus and determine the temperature needed to elicit moderate pain in each participant. Each test consisted of 10 heat contacts, 1.5 second duration, with onsets every 3 seconds over the span of 30 s with the thermode temperatures ranging between 40 and 52 °C (the temperature was constant during each test). This stimulation paradigm can cause wind-up, and the time interval between the contacts produces a robust BOLD response without receptor adaptation. We chose this paradigm because FM is believed to involve central sensitization, which is exacerbated by a wind-up paradigm. Prior studies using a thermal stimulus have shown that participants with FM have altered responses to this paradigm compared to pain-free participants (7, 8, 10). In addition, the choice of a thermal stimulus allows for the calibration of pain intensity for each participant's level of sensitivity and can be easily applied in an MRI environment.

Each participant received the same stimulus intensities in the same order, consisting of trials of 46, 50, 44, and 48°C respectively. During each test, participants verbally rated each of the 10 contacts out loud using the NPS. They were encouraged to rate in increments of 5 but were not corrected if they used other numbers. The temperatures used never exceeded 52°C to prevent tissue damage. Participants were kept blinded to this objective as well as to the temperatures used during the tests to avoid any response bias. They were informed that if the sensation was ever intolerable they could remove their hand from the device at any time during the study. This served both to relieve their anxiety as well as avoid causing high levels of subjective pain.

The training session concluded with a practice run of the experimental protocol in the sham MRI scanner. To prevent motion artifacts, participants cannot verbalize their ratings during the imaging, therefore they were instructed to rate each contact mentally and remember the ratings they gave for the first and last contact. The sham tests also allowed participants to practice laying as still and relaxed as possible to avoid movement during imaging, and they were reassured that the NPS would be displayed, requiring no memorization of the scale. The sham MRI provides an environment similar to the MRI to allow participants to familiarize themselves with how imaging will feel and ease anxiety. Participants lay supine on a mobile bed and were provided with a mirror over their eyes to view a rear-projection screen and listen to recorded sounds from MRI scans that were played for them on a speaker. The practice scan for this study used the same stimulation paradigm as the subsequent imaging session, allowing participants to practice mentally rating their pain and recalling the first and last ratings.

### FMRI Paradigm

This study employed a “threat vs. safety” paradigm to allow us to examine periods of anticipation of pain, periods of painful stimulation, and periods of rest. The imaging session consisted of 10 fMRI runs of 4.5 min each, separated into five “Pain” runs in which participants experienced the noxious heat stimulus, interleaved in a randomized order with five ‘No-Pain' runs in which participants did not feel the stimulus. While the majority of participants completed this session, some participants only underwent four “Pain” runs due to time constraints or participants being unable to comfortably lie still for the amount of time needed to complete all five runs. During each run, at the 1-minute mark, they were informed via a rear-projection screen whether they would feel the stimulus or not that run. If it was a “Pain” run, participants were told at the 2-min mark that the stimulation would begin. During stimulation, they experienced 10 heat contacts over a span of 30 seconds at the calibrated temperature. During the stimulation, the NPS was displayed and participants were instructed to mentally rate each contact on the scale. After the stimulation, imaging continued for another 2 min before ending. After each “Pain” run, participants were asked over an intercom to give their ratings for the first and last contact, and were told that another run would begin soon. Imaging was conducted over the same amount of time in the No Pain condition, but participants were told they would not receive the stimulus. This paradigm has been previously employed in several pain studies (22, 27, 28, 49).

### FMRI Data Acquisition

This study included data from a larger research program that included both brainstem/spinal cord and brain imaging sessions. Only the brainstem/spinal cord imaging data are discussed here. Functional MRI scans were carried out on a Siemens 3 tesla MRI system (Siemens Magnetom, Erlangen, Germany). During the data collection phase of this study, the MR system underwent an upgrade from a Siemens Magnetom Trio to a Siemens Magnetom Prisma. Efforts were made to keep scan quality equivalent pre- and post-upgrade, and checks were performed with data from the FM and HC groups before and after the upgrade as well as additional volunteer data to compare the quality of the data and the signal to noise ratio in each. No significant differences were found in scan quality or signal-to-noise ratio before and after the upgrade.

Localizer images were acquired in three planes to provide a reference for the subsequent slice positions. Functional images were acquired using a half-Fourier single-shot fast spin-echo (HASTE) sequence with BOLD contrast, spanning the full brainstem and cervical spinal cord (first thoracic vertebra to above the thalamus). This method has been shown to provide optimal image quality and BOLD sensitivity in the brainstem and spinal cord (48). The 3D volume was imaged in 9 contiguous sagittal slices, 2 mm wide, with a 28 × 21 cm field-of-view and a 1.5 × 1.5 mm in-plane resolution. Imaging parameters included an echo time (TE) of 76 ms and a repetition time (TR) of 6.75 s/volume for optimal T2-weighted BOLD sensitivity. Each imaging run consisted of 40 volumes (equivalent to a 4.5 min run). In total, 10 runs were acquired for each participant, 5 Pain and 5 No Pain, therefore each condition consisted of 200 volumes per individual.

### FMRI Data Preprocessing and Analysis

#### Data Preprocessing

Preprocessing was carried out using custom-written software (48), “spinalfmri9” (https://www.queensu.ca/academia/stromanlab/home/fmri-analysis-software) in MATLAB (MathWorks, Natick, MA, USA). Image data were first converted from DICOM to NIfTI format, after which they were co-registered to correct for bulk body motion using the non-rigid 3D registration tool in the MIRT package (Medical Image Registration Toolbox) (50, 51). Images were then resized to 1 mm^3^ voxels and spatially normalized to a pre-defined anatomical template, as described previously (27, 47). Physiological noise estimates were obtained from the recording of the peripheral pulse (synchronized to each fMRI time series), estimates of global noise were obtained from regions of white matter, and motion parameters obtained from the co-registration procedures were used as models of bulk movement. These noise models were fit to the data using a general linear model (GLM) and subtracted from the data. This method has been shown to be highly effective for removing physiological noise in this region (52).

#### Data Analysis

The threat/safety paradigm enables comparisons of periods with and without the anticipation, and experience, of the noxious heat stimulus. For this study we focused the analysis on two time periods; the second minute of the baseline period preceding the noxious stimulus, and the stimulation period. Two epochs were analyzed, consisting of 45 s blocks. The first epoch is centered around the 1 min 30 s mark and is termed the “Expectation” period. This is the time after participants were told to expect pain, but before they had experienced any stimulus. The second epoch is centered around the 2 min 15 sec mark and is termed the “Stimulation” period, and is the time when participants were experiencing a painful stimulus. This also allowed us to compare these results to our previous studies (28, 30).

Analyses consisted of characterizations of BOLD responses, and connectivity analyses. As it is not practical to apply these analyses on a voxel-by-voxel basis, we selected 10 regions of interest (ROIs) in the brainstem and spinal cord which were identified using a previously-established anatomical region map (7, 47, 53). These regions and their expected locations were compiled from several anatomical atlases and published articles (54–58). The regions included the thalamus, hypothalamus (Hyp), periaqueductal gray matter (PAG), parabrachial nucleus (PBN), locus coeruleus (LC), nucleus tractus solitarius (NTS), nucleus raphe magnus (NRM), nucleus gigantocellularis (NGc), dorsal reticular nucleus of the medulla (DRt), and the pontine reticular formation (PRF). As the noxious stimulus was applied to the palm of the right hand, which corresponds to the C6 dermatome, we also included the right dorsal quadrant of the 6^th^ cervical spinal cord segment (C6RD). However, because entire anatomical regions are not expected to be uniformly involved with pain responses (28, 30, 34, 59, 60), regions were divided into sub-regions based on fMRI time-course properties. Each ROI was divided into 5 clusters of voxels (i.e., sub-regions) by means of k-means clustering. This method provides greater spatial precision by dividing the voxels into clusters based on their functional characteristics.

#### Structural Equation Modeling

**Figure 1:**
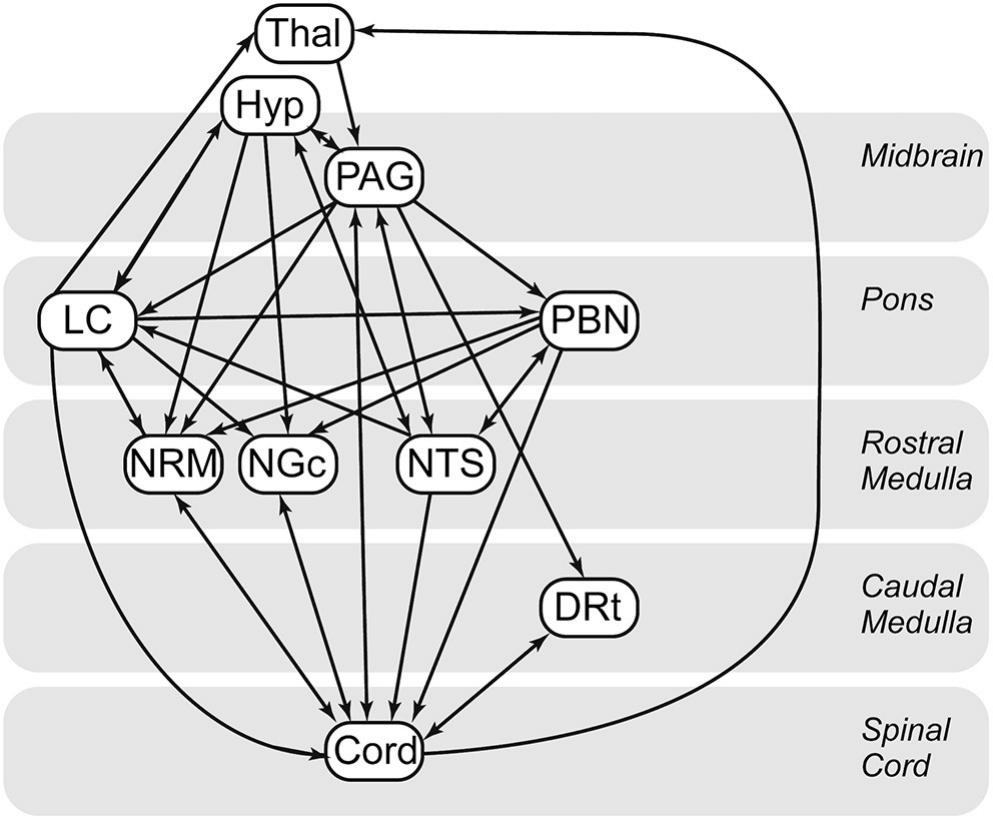
Anatomical model of the regions and connections used for the structural equation modeling (SEM) analysis.

Cluster-to-cluster correlations may not always be sufficient to explain complex coordination between regions (28, 30, 59). As in previous studies (27, 28, 30, 49, 59), we used structural equation modeling to examine coordinated networks. These methods have been validated for use in the brainstem and spinal cord (61), and in previous studies they have been used to identify and characterize robust networks in the brain, brainstem and spinal cord (30, 59), as well as characterize connectivity networks during pain processing (60) and the expectation of pain (28). SEM requires a pre-defined model of anatomical regions and connections between these regions. We have chosen a previously-described model based on known pain related neuroanatomy (23, 62–64) (Figure 1), which includes the brainstem regions described above, the C6RD quadrant of the spinal cord, as well as information about the anatomical directionality of connections.

Connectivity analyses (SEM) were carried out separately for the Expectation and Stimulation periods by means of a general linear model (GLM) fitting method which was used to calculate the linear weighting factors (β) which describe the relative contribution of each “source” input to a “target” region. These β values reflect the connectivity strength between regions, and are calculated as follows: if a region A receives inputs from two other regions, B and C, and the BOLD signal time series in these responses are S_A_, S_B_, and S_C_ respectively, then S_A_ = β_AB_S_B_ + β_AC_S_C_ + e_A_ where e_A_ is the residual signal variation that is not explained by the fit. Within the model are several network components that consist of a target region (e.g., S_A_) and the multiple source regions providing input to that target region (e.g., S_B_ and S_C_). The weighting factors (beta, β) were calculated separately for each network component, and networks were investigated for every combination of clusters of each region in order to identify the clusters that resulted in the best fits to the data measured. The beta value for each connection is therefore calculated several times with different combinations of “source” and “target” clusters. The amount of variance in each target region that can be explained by the fit was calculated and expressed as an R^2^ value, and the significance of the fit was estimated by converting R values to a Z-score by means of a Fisher's Z-transform. This fitting was repeated with one source region at a time omitted from the network, and an F-test was used to identify terms that did not uniquely account for a significant component of the variance in each target region. Any terms that did not account for a significant component of the target region variance were not included in the results. A threshold of F(1,∞) > 3.845 was used to determine significance (corresponding with *p* < 0.05).

#### Analyses and Comparisons of Connectivity Networks

Connectivity networks were compared between and within the FM and HC groups, for each time period, by means of analyses of covariance (ANCOVA). The ANCOVA included the Group (FM vs. HC) as a discrete variable, and the participants' normalized pain scores, as a continuous variable. Normalized pain scores were calculated for each individual by taking the ratio of their average pain rating during the “Pain” runs to the average temperature applied to the hand (i.e., pain rating/temperature). A higher ratio reflects higher pain sensitivity. The results thus demonstrate significant group differences, significant dependences on pain ratings, and interactions between the group and pain ratings. Significance was inferred at a multiple-comparison corrected probability threshold of *p* < 0.05, using a Bonferroni family-wise error-rate correction to account for the number of independent connections tested. ANCOVA analyses were applied separately using data in the “Expectation” and “Stimulation” periods.

## Results

### Participant Characteristics

Questionnaire scores were compared between study groups (FM vs. HC) by means of Student's *T*-tests, and are summarized in Tables 1, 2. Fibromyalgia participants were observed to have a significantly higher normalized pain scores than healthy controls, *t*(28) = −3.303, *p* = 0.003. They also scored significantly higher on measures of depression (BDI), measures of pain catastrophizing (total and sub-scores of rumination, magnification, and helplessness), measures of pain symptomatology (total FIQR and function, impact and symptom subscales), measures of autonomic function (total COMPASS and all subscales), and pain inventory scores (total MPQ and continuous, intermittent, neuropathic, and affective subscales). No demographic information such as age, smoking habits, or drinking, was significantly different between the groups.

**Table 1:** Demographic information for the healthy control (HC) and fibromyalgia (FM) groups. Where applicable the mean score is given, followed by the standard deviation in parentheses. The normalized pain score was calculated by dividing each individual's average pain rating by the average stimulus temperature needed to elicit that rating. A higher number indicates higher pain sensitivity. The participant groups had significantly different scores on all four measures.

**Demographic information**	**HC (SD)**	**FM (SD)**
Age	39.2 (10.3)	46.7 (13.5)
BMI	27.6 (3.8)	25.8 (5.1)
Normalized pain score	0.72 (0.2)	1.01 (0.2)
Initial pain score	2.3 (5.62)	33.9 (23.7)

**Table 2:** Significant differences in group means (FM vs. HC) of questionnaire scores. Group means, standard error, t value, and p value are given for each comparison. All comparisons listed show significant differences between the groups. Other differences that were tested but were not significant were state-trait anxiety and social desirability. Acronyms, in order given in the table, represent questionnaire scores for depression (BDI), pain catastrophizing (PC) including total questionnaire scores and subscales, fibromyalgia impact (FIQR) including total scores and subscales, autonomic symptoms (COMPASS) including total scores and subscales for various symptom categories, normalized pain score (calculated as the ratio of the average pain rating given to the average temperature of the stimulus), and pain symptoms (MPQ) including total scores and subscale scores. The initial pain rating refers to the rating participants gave for their overall bodily pain before starting the study, using the same 100 point scale they were trained to use during the sham MRI session.

**Questionnaire**	**HC (SE)**	**FM (SE)**	**t**	***p***
BDI	7.13 (2.45)	16.26 (2.77)	−2.462	0.02
PC total	6.71 (1.7)	21.28 (3.29)	−3.930	0.001
PC rumination	3.57 (0.91)	7.64 (1.25)	−2.629	0.014
PC magnification	1.0 (0.31)	4.21 (0.68)	−4.286	0.001
PC helplessness	2.14 (0.67)	9.42 (1.71)	−3.963	0.001
FIQR total	10.31 (3.18)	50.26 (3.66)	−8.223	0.001
FIQR function with FM	1.11 (0.67)	11.22 (1.53)	−6.040	0.000
FIQR impact of FM	1.26 (0.78)	10.0 (1.02)	−6.756	0.000
FIQR symptoms of FM	7.93 (1.92)	29.03 (1.63)	−8.370	0.000
COMPASS total	12.96 (2.36)	39.53 (4.31)	−5.395	0.000
COMPASS orthostatic intolerance	5.6 (1.78)	14.94 (2.74)	−2.855	0.008
COMPASS vasomotor	0.0 (0.0)	1.93 (0.37)	−5.263	0.000
COMPASS secretomotor	1.28 (0.54)	7.02 (0.94)	−5.246	0.000
COMPASS gastrointestinal	4.7 (0.80)	10.58 (1.21)	−4.047	0.000
COMPASS bladder	0.44 (0.23)	2.44 (0.77)	−2.461	0.02
COMPASS pupillomotor	0.94 (0.24)	2.56 (0.26)	−4.554	0.000
Normalized pain score	0.71 (0.59)	1.01 (0.06)	−3.303	0.003
Initial pain	2.33 (5.62)	33.92 (23.77)	−4.848	0.000
MPQ total	12.8 (3.66)	87.13 (12.1)	−5.88	0.00
MPQ continuous	5.5 (1.36)	28.93 (3.26)	−6.614	0.00
MPQ intermittent	2.0 (1.18)	21.67 (4.32)	−4.383	0.000
MPQ neuropathic	3.4 (1.9)	22.80 (3.81)	−4.551	0.000
MPQ affective descriptors	1.9 (1.17)	13.73 (2.16)	−4.796	0.000

*Shaded cells represent overall questionnaire scores, while unshaded cells represent subscales of the questionnaires*.

To examine the relationship between traits such as pain catastrophizing, anxiety, depression, autonomic function, and pain symptomatology and experience, Spearman's rank correlations of all scale and subscale scores with normalized pain scores was also conducted (Table 3). This was done separately for FM and HC participants. In the FM group, age was significantly correlated with pain scores, with older participants having higher pain scores, *rho*(13) = 0.567, *p* = 0.02. No other correlations were found to be significant.

**Table 3:** Pearson's correlations of group questionnaire scores (FM or HC) with individual normalized pain score (calculated as mean pain intensity/mean stimulus temperature for each individual).

	**Healthy controls**	**Fibromyalgia**
**Questionnaires**	**Normalized pain score (rho)**	**Normalized pain score (rho)**
Age	−0.084	**0.567**
BMI	−0.038	−0.347
Anxiety (STAI)	0.028	−0.426
Depression (BDI)	−0.020	−0.347
PC total	−0.063	0.425
PC rumination	−0.128	0.172
PC magnification	−0.034	0.426
PC helplessness	−0.025	0.513
FIQR total	−0.260	0.195
FIQR function with FM	−0.110	0.220
FIQR impact of FM	−0.110	0.074
FIQR symptoms of FM	−0.246	0.081
COMPASS total	−0.055	0.093
COMPASS orthostatic intolerance	−0.122	−0.128
COMPASS vasomotor	NA	0.078
COMPASS secretomotor	0.170	0.072
COMPASS gastrointestinal	−0.027	0.318
COMPASS bladder	−0.485	0.314
COMPASS pupillomotor	−0.154	0.245
MPQ total	−0.280	0.257
MPQ continuous	0.055	0.272
MPQ intermittent	−0.270	0.094
MPQ neuropathic	−0.311	0.277
MPQ affective descriptors	−0.216	0.183

*Spearman's rank rho values are given for each comparison, with any values significant at p < 0.05 given in bold font. Acronyms, in order given in the table, represent questionnaire scores for depression (BDI), pain catastrophizing (PC) including total questionnaire scores and subscales, fibromyalgia impact (FIQR) including total scores and subscales, autonomic symptoms (COMPASS) including total scores and subscales for various symptom categories, normalized pain score (calculated as the ratio of the average pain rating given to the average temperature of the stimulus), and pain symptoms (MPQ) including total scores and subscale scores. Shaded cells represent overall questionnaire scores, while unshaded cells represent subscales of the questionnaires. Note that the COMPASS Vasomotor cell in the healthy control group has no rho value. This is because all healthy controls scored zero points on this COMPASS subscale (no impairment) and a correlation could therefore not be computed with one variable being a constant*.

### Network Comparisons

SEM analyses identified extensive networks in the brainstem and spinal cord for both participant groups, in the Expectation and Stimulation periods. Table 4 summarizes the significant connectivity values in both groups during both periods of interest. While expecting pain, SEM results from both groups included significant connectivity from the LC to the PBN and from the PAG to the LC. Significant network connectivity was also observed from the LC and the PAG to the NGC in the HC group, while significant hypothalamus to LC and PAG to PBN connectivity was observed in the FM group. During the stimulation period, both groups had significant PAG to LC network connectivity. The connectivity network observed in the healthy control group also included connections from the LC to the PBN and thalamus, from the hypothalamus to the NTS, from the thalamus to the PAG, and from the NTS to the LC. In contrast, the network connectivity observed in the FM group during the painful stimulation also included connections from the PAG to the PBN and DRt, and from the hypothalamus to the NRM.

**Table 4:** Summary of significant spinal cord/brainstem connectivity in the healthy control (first column) and fibromyalgia (second column) groups, analyzed with SEM. The upper section of the table summarizes connectivity during the Expectation period, and the lower section summarizes the Stimulation period.

**Healthy controls**		**Fibromyalgia**	
**Region Source → Target**	**β** **±SE**	**Region Source → Target**	**β** **±SE**
**Expecting pain**		**Expecting pain**	
LC → PBN	0.13 ± 0.03	LC → PBN	0.13 ± 0.02
LC → PBN	0.17 ± 0.04	LC → PBN	0.10 ± 0.02
PAG → LC	0.42 ± 0.03	PAG → LC	0.39 ± 0.05
LC → NGC	0.27 ± 0.05	Hypothalamus → LC	0.32 ± 0.05
PAG → NGC	0.22 ± 0.05	PAG → PBN	0.18 ± 0.04
**Experiencing pain**		**Experiencing pain**	
PAG → LC	0.29 ± 0.07	PAG → LC	0.39 ± 0.04
LC → PBN	0.18 ± 0.04	PAG → PBN	0.25 ± 0.05
LC → Thalamus	0.09 ± 0.02	PAG → PBN	0.19 ± 0.05
Hypothalamus → NTS	0.27 ± 0.06	Hypothalamus → NRM	0.44 ± 0.09
Thalamus → PAG	0.42 ± 0.09	PAG → DRt	0.30 ± 0.09
NTS → LC	0.28 ± 0.06	PAG → PBN	0.25 ± 0.04

*For each connection, the β value and standard error calculated with SEM are given. All connections listed have statistically significant β values. Repeated connections between the same regions indicate that different clusters within the regions had significant connectivity. Abbreviations: dorsal reticular nucleus (DRt), locus coeruleus (LC), nucleus raphe magnus (NRM), nucleus gigantocellularis (NGc), nucleus tractus solitarius (NTS), periaqueductal gray (PAG), parabrachial nucleus (PBN), and the right dorsal region of the 6^th^ cervical cord segment (C6RD)*.

The results of ANCOVA analyses to compare connectivity values between groups, and in relation to pain scores, are listed in Table 5. The results show significant main effects of the study group and pain scores as well as significant interaction effects in both the Expectation and Stimulation time periods. During the Expectation time period, there were significant main effects of the study group in connections from the hypothalamus to the NRM and NGc, from the PAG to the NGc, and from the spinal cord to the thalamus. During the Stimulation period, these effects were observed in connections from the LC to the NRM, from the PAG to the LC, NGc, and hypothalamus, from the spinal cord to the NGc and PAG, and from the PBN to the NGc. Connectivity between regions also varied significantly with normalized pain scores in both time periods. In the Expectation period, this connectivity was localized within brainstem regions (from the hypothalamus to the LC, and from the PAG to the LC, and NGc). In the Stimulation period, these effects were relatively similar in terms of brainstem-to-brainstem connectivity, but also included additional connections from the PBN and spinal cord to the NGc, and from the hypothalamus and LC to the NRM. Interaction effects were more pronounced in the Expectation time period, and involved connectivity from the spinal cord to the thalamus, from the PAG and LC to the NGc, from the PAG and hypothalamus to the NRM, and from the NTS to the LC. In contrast, interaction effects in the Stimulation period involved only brainstem to brainstem connectivity, namely from the PAG to the PBN and hypothalamus, and from the PBN to the NGc.

**Table 5:** ANCOVA results for both the Expectation and Stimulation epochs, comparing main effects of group (FM vs. HC), main effects of normalized pain scores, and group x pain score interaction effects.

**Expecting pain**	**Experiencing pain**
**Region Source → Target**	**Region Source → Target**
**Main effect of Group** **(FM vs. HC)**	**Main effect of Group (FM vs. HC)**
PAG → NGC	LC → NRM
C6RD → Thalamus	C6RD → PAG
Hypothalamus → NGC	PAG → LC
Hypothalamus → NRM	PBN → NGC
	C6RD → NGC
	PAG → Hypothalamus
	PAG → NGC
**Main effect of Pain Score**	**Main effect of Pain Score**
PAG → NGC	PAG → LC
Hypothalamus → LC	PBN → NGC
PAG → LC	Hypothalamus → NRM
	LC → NRM
	C6RD → NGC
	PAG → NGC
**Interaction effect** **(Group x Pain Score)**	**Interaction effect (Group x Pain Score)**
C6RD → Thalamus	PAG → PBN
PAG → NGC	PBN → NGC
LC → NGC	PAG → Hypothalamus
PAG → NRM	
Hypothalamus → NRM	
PAG → NGC	
NTS → LC	

*The table summarizes all statistically significant effects*.

## Discussion

The results of this study demonstrate important differences in pain processing between people with FM and a healthy control group. The differences exist across participant characteristics, pain behavioral responses, and coordinated brainstem/spinal cord function identified by means of fMRI, and they demonstrate that altered pain processing in FM may be linked to changes in both descending pain regulation and autonomic regulation. This study is also the first to show that these differences in FM are present *before* a noxious stimulus is applied, while the participants are anticipating the pain.

Our SEM analyses confirm that extensive brainstem and spinal cord network connectivity exists during the expectation and experience of pain in both control participants and women with fibromyalgia (Table 4). Both groups showed extensive connectivity between the LC, PAG, and PBN brainstem regions both while expecting and experiencing pain. The PAG is a key brainstem region associated with descending modulation of pain (23), while the LC and PBN have functions associated with pain modulation, motivational affective aspects of pain, as well as autonomic homeostatic regulation (28, 60, 65). Connectivity between these regions has been previously identified in other studies both during the expectation and experience of pain in healthy controls (28, 49). Importantly, these regions were also part of key elements identified as part of the brainstem networks associated with the expectation of pain specifically (28).

**Figure 2:**
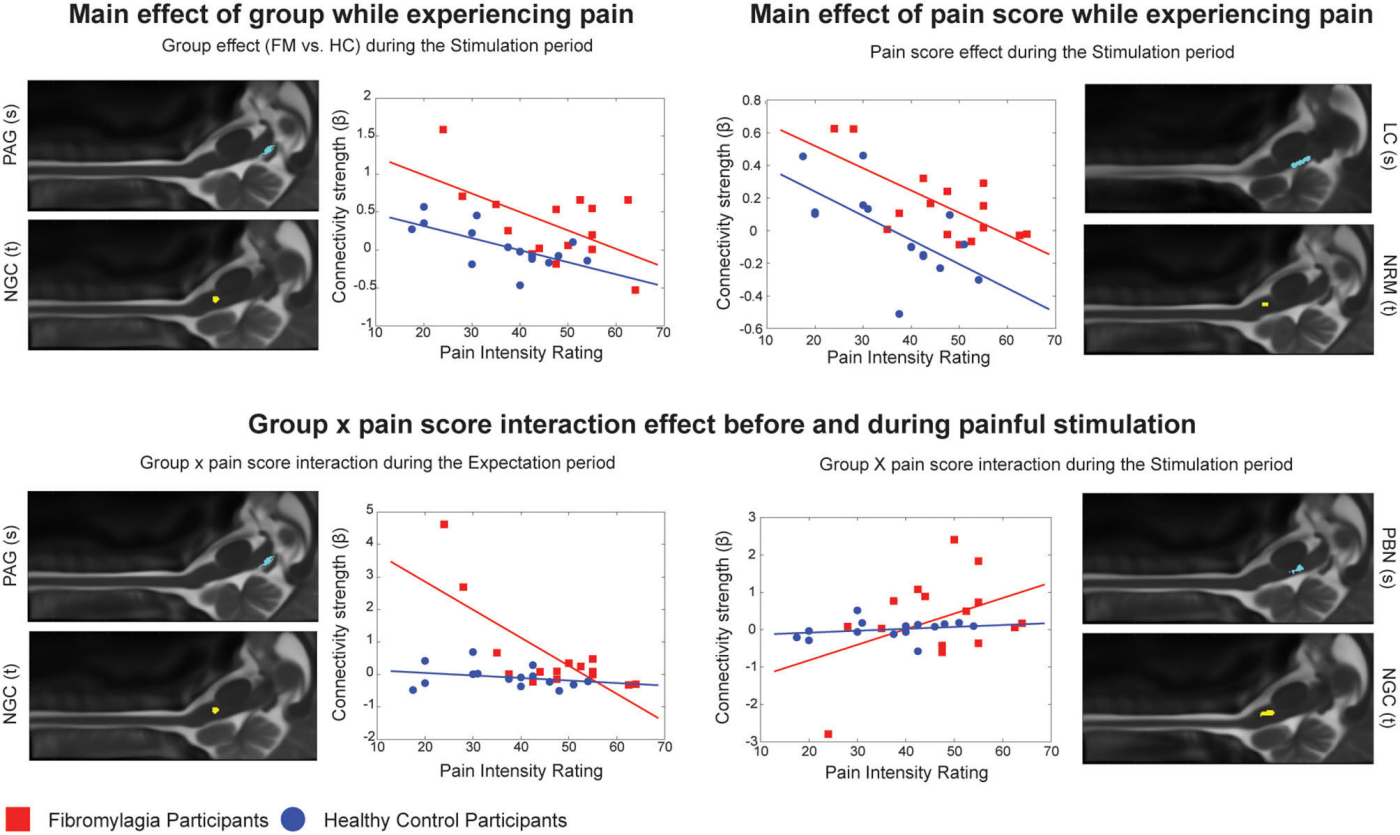
Connection details for 4 example connections obtained from ANCOVA results for both the Expectation and Stimulation periods, comparing main effects of group (FM vs. HC), main effects of normalized pain score, and group x pain score interaction effects. Source regions are denoted with (s) while target regions are denoted with (t). Red points represent individual participants with fibromyalgia while blue points represent the healthy controls. For each figure, the x axis shows the individual average pain intensity ratings for the stimulus while the y axis shows the individual connectivity strengths (calculated and represented as a β value).

Comparisons between the groups (ANCOVA) also revealed significant differences in pain processing between fibromyalgia participants and healthy control women, both before and during painful stimulation (Table 5). Figure 2 shows details of relationships between connectivity strengths and pain scores for selected connections with significant main effects of group, normalized pain scores, and interaction effects. The selected connections include an example of a main effect of the Group (FM vs. HC) in the PAG to the NGc connection, in the Expectation period. The connectivity values varied with pain scores in both groups, but had consistently higher values in FM. These results support previous evidence that FM may involve altered descending regulation (11), and show that this is the case even when differences in individual pain scores are taken into account.

Furthermore, we were able to demonstrate that such differences in brainstem and spinal cord connectivity exist between fibromyalgia and healthy control participants both before and during the painful stimulation (Table 5). In the Expectation period, group differences in connectivity involved mainly signaling from the hypothalamus to brainstem areas such as the NGc and NRM, and feedback signaling from the spinal cord to the thalamus. This may indicate that a component of fibromyalgia pain is altered pain modulation during the anticipation of pain. As we demonstrated in our previous study, pain modulation includes a continuous component which is present before a painful stimulus is applied and may contribute to readying spinal cord areas to receive incoming nociceptive signals (49). If this process is altered in fibromyalgia, it may explain why these participants often have disproportionate pain responses to similar stimuli as given to healthy controls, or why they require stimuli of lower intensity to elicit similar pain responses to healthy controls (including in the current study) (7, 8, 11–20).

In contrast, differences in the Stimulation period involve more extensive brainstem to brainstem signaling as well as some feedback signaling from the spinal cord to brainstem areas such as the PAG and NGc. These results support our expectations that descending pain regulation is altered in fibromyalgia during noxious stimulation, as the primary differences in connectivity involve the PAG-RVM-spinal cord descending pain modulation pathway (23). However, there are additional connectivity differences involving areas such as the LC, the hypothalamus and the PBN, whose function is associated in part with arousal and autonomic regulation (34). The areas involved in these differences may therefore indicate that this altered descending regulation may have a contribution from altered autonomic signaling. While FM pain has been associated with autonomic dysfunction in previous behavioral studies (31–33), this is the first study to show evidence of this link that is supported by fMRI data.

The ANCOVA analysis also showed a significant main effect of normalized pain scores in both the Expectation and Stimulation time periods, indicating that pain scores are linked to connectivity strengths in these networks regardless of which participant group the participants were in (Figure 2). During the Expectation period these differences were exclusively signaling to and from brainstem areas including the PAG and LC, and did not involve any significant connectivity to/from the spinal cord. In a previous study, we demonstrated that several components of these brainstem and spinal cord networks may be specific to expecting pain (28), and many of these components are seen here in this brainstem-to-brainstem signaling as varying with individual pain scores. These connections were also part of significant group x pain score interaction effects, where the observed connectivity strengths depended on a combination of the participants' normalized pain scores as well as which group they belonged to. These results indicate that the activity changes in these regions and the differences in pain scores in fibromyalgia are closely linked.

One review of chronic pain studies suggests a link between certain types of emotional regulation and altered pain responses (66). The authors showed that maladaptive emotional regulation in response to acute pain may contribute to depressed mood and enhanced pain catastrophizing (but not anxiousness), and in turn mediate altered pain responses. Our results align with this idea as we found that women with fibromyalgia had higher BDI and PCS scores than the controls but not STAI scores (Table 2), even though both anxiety and depression are known to be comorbid with the condition (1, 67–69). A recent study also showed that people with fibromyalgia have more difficulty regulating emotions, and this predicted heightened pain responses depending on their coping strategies (70). Some brain fMRI studies in pain-free people identified that connectivity changes between the PAG and some cortical areas were associated with a participant's tendency to disengage their attention from the pain and obtain pain relief through distraction (71, 72). Our fibromyalgia group had significantly higher pain catastrophizing scores than the healthy control group, a measure which takes into account in part how people think about and attend to their pain (40). It is possible that a similar process is occurring here with connectivity between the PAG and other brainstem areas.

While we have no direct measures of emotional regulation and coping strategies in the current study, we do have evidence that the altered pain experienced by the fibromyalgia participants is likely due to alterations in a convergence of autonomic regulation and pain modulation systems. Women with fibromyalgia also scored higher on the COMPASS-31 questionnaire which measures symptoms of autonomic system dysfunction. These symptoms may be related to the increased pain sensitivity that is a characteristic of fibromyalgia (1, 2, 73–75), as previous studies have also noted that HPA axis dysregulation may be a part of chronic pain symptomatology and manifestation (33, 76–79). As our results show that connectivity between the PAG and other brainstem areas varies with a participant's pain score, it is possible that this link may be driving some of the differences in pain processing we observed in the fibromyalgia group (who, on average, have higher normalized pain scores than the healthy controls). Motivational-affective components of pain processing and autonomic control are closely interlinked and have been shown to contribute to altered pain responses in the brain (12–15). Based on this and the evidence that maladaptive emotional regulation (an affective component of pain processing) can lead to altered pain in fibromyalgia (66, 70), it is possible that our results show the underlying neural basis of these effects at the level of the brainstem and spinal cord.

### Limitations

This article uses SEM as a hypothesis-driven and data-driven analytical approach to fMRI pain data. Structural equation modeling requires a pre-defined anatomical model and can therefore not give information on other regions present in the spinal cord and brainstem that were not included in the original network. Our network was chosen to include regions known to be associated with pain and pain modulation, homeostatic regulation, and arousal. While we are confident in the results presented, we cannot guarantee that other effects are not present in other regions which may influence the connectivity changes in the given network. There were also unintended differences in the age of participants although efforts were made to age-match participants wherever possible. Lastly, it must be noted that FM is a heterogenous condition with a spectrum of possible symptoms and presentations. Our results are an important step for exploring overall pain processing differences between FM and healthy controls, but more studies are needed to expand on this and explore how these results generalize to different FM populations with different symptom presentations.

### Conclusions

These results are an important step in advancing our understanding of fibromyalgia. Women with fibromyalgia have altered descending pain modulation compared to healthy controls. Furthermore, these differences can exist without a noxious stimulus, as network connectivity in the brainstem and spinal cord is altered during both the expectation and the experience of pain. Importantly, many of these changes in network connectivity in FM were related at least in part to individual normalized pain scores. While many brainstem areas carry out several different functions, the areas involved in these connectivity differences seem to indicate that altered pain in fibromyalgia may be the result of changes in a convergence of systems involved with pain regulation, arousal, and autonomic homeostatic regulation. The latter is especially interesting, as links of fibromyalgia pain with changes in autonomic system function have been demonstrated previously by some important behavioral research, which can now be in part supported with novel findings from brainstem and spinal cord fMRI data. Our evidence supports the conclusion that fibromyalgia may involve changes in how autonomic regulation is integrated with descending pain regulation in the brainstem and spinal cord.

## Data Availability Statement

The data that support the findings of this study are available from the corresponding author upon reasonable request.

## Ethics Statement

The studies involving human participants were reviewed and approved by Queen's University Research Ethics Board. The patients/participants provided their written informed consent to participate in this study.

## Author Contributions

GI: participant recruitment, data collection, data analysis, data interpretation, first manuscript preparation, and manuscript review. HW and JP: participant recruitment, data collection, data analysis, data interpretation, and manuscript review. RS and CP: data interpretation and manuscript review and editing. PS: study design, data collection, data analysis, data interpretation, and manuscript review and editing. All authors contributed to the article and approved the submitted version.

## Funding

This research was funded by the National Sciences and Engineering Research Council (NSERC), RGPIN/06221-2015. This work was also supported by Spectrum Therapeutics through MITACS.

## Conflict of Interest

The authors declare that the research was conducted in the absence of any commercial or financial relationships that could be construed as a potential conflict of interest.

## Publisher's Note

All claims expressed in this article are solely those of the authors and do not necessarily represent those of their affiliated organizations, or those of the publisher, the editors and the reviewers. Any product that may be evaluated in this article, or claim that may be made by its manufacturer, is not guaranteed or endorsed by the publisher.
